# A systematic review and meta-analysis of the safety and efficacy of left atrial substrate modification in atrial fibrillation patients with low voltage areas

**DOI:** 10.3389/fcvm.2022.969475

**Published:** 2022-09-20

**Authors:** Shaobin Mao, Hongxuan Fan, Leigang Wang, Yongle Wang, Xun Wang, Jianqi Zhao, Bing Yu, Yao Zhang, Wenjing Zhang, Bin Liang

**Affiliations:** ^1^Graduate school of Shanxi Medical University, Taiyuan, China; ^2^Department of Cardiovascular Medicine, Second Hospital of Shanxi Medical University, Taiyuan, China

**Keywords:** atrial fibrillation, catheter ablation, low-voltage areas, recurrence, meta-analysis

## Abstract

**Background:**

The left atrial low-voltage areas (LVAs) are associated with atrial fibrosis; however, it is not clear how the left atrial LVAs affect the recurrence of arrhythmias after catheter ablation, and the efficacy and safety of the left atrial substrate modification based on LVAs as a strategy for catheter ablation of atrial fibrillation (AF) are not evident for AF patients with LVAs.

**Methods:**

We performed a systematic search to compare the arrhythmia recurrence in AF patients with and without LVAs after conventional ablation and arrhythmia recurrence in LVAs patients after conventional ablation with and without substrate modification based on LVAs.

**Result:**

A total of 6 studies were included, involving 1,175 patients. The arrhythmia recurrence was higher in LVA patients after conventional ablation (OR: 5.14, 95% CI: [3.11, 8.49]; *P* < 0.00001). Additional LVAs substrate modification could improve the freedom of arrhythmia in LVAs patients after the first procedure (OR: 0.30, 95% CI: [0.15, 0.62]; *P* = 0.0009). However, there was no significant difference after multiple procedures (*P* = 0.19). The procedure time (MD: 26.61, 95% CI [15.79, 37.42]; *P* < 0.00001) and fluoroscopy time (MD: 6.90, 95% CI [4.34, 9.47]; *P* < 0.00001) in LVAs patients with additional LVAs substrate modification were significantly increased compared to LVAs patients’ without substrate modification. Nevertheless, there were no higher LVAs substrate modification-related complications (*P* = 0.93) between LVAs patients with and without additional LVAs substrate modification. In the subgroup analysis, the additional LVAs substrate modification reduced the risk of arrhythmia recurrence in LVAs patients during the follow-up time, which was 12 months (OR: 0.32, 95% CI (0.17, 0.58); *P* = 0.002), and box isolation (OR: 0.37, 95% CI (0.20, 0.69); *P* = 0.002) subgroups, but the type of AF, follow up >12 months and homogenization subgroups were not statistically significant. Trial sequential analysis shows conclusive evidence for the LVAs ablation.

**Conclusion:**

This study has shown that LVAs could improve the risk of arrhythmia recurrence in AF patients after conventional ablation. And additional LVAs substrate modification after conventional ablation could increase the freedom of arrhythmia recurrence in LVAs patients. Interestingly, the box isolation approach appeared more promising.

**Systematic review registration:**

[http://www.crd.york.ac.uk/prospero], identifier [CRD42021239277].

## Introduction

Catheter ablation is an effective strategy for rhythm control of atrial fibrillation (AF) (1, 2). The procedure of pulmonary vein isolation (PVI) is the cornerstone of catheter ablation for all types of AF. However, the PVI alone has reported recurrence rates as high as 40% within one year (3). This may be because triggers are not limited in pulmonary veins but also appear in other left atrial substrates, especially in persistent AF (4). Previous studies have found that the left atrial low-voltage areas (LVAs), as left atrial substrates, are independent predictors of recurrence after PVI (5–7). In addition, LVAs have been reported to be associated with atrial fibrosis which can lead to conduction slowing and arrhythmia, as verified by late gadolinium enhancement (LGE) magnetic resonance imaging (8–10). Therefore, in order to improve freedom for AF arrhythmia, the voltage mapping-guided LVAs substrate modification could be an established ablation strategy to eliminate the LVAs arrhythmic substrate. It has been shown that left atrial substrate modification based on LVAs has superb application prospects in many previous studies (11–14). However, some studies have found inconsistent results (15–17).

The systematic review and meta-analysis synthesize the limited data regarding the left atrial substrate modification by targeting LVAs ablation, and attempt to determine whether this ablation strategy is more superior in LVAs patients.

## Methods

The systematic review and meta-analysis was conducted using the guidelines described in the Preferred Reporting Items for Systematic.

Reviews and Meta-Analysis (PRISMA) (18), and registered with International Prospective Register of Systematic Reviews (PROSPERO).

### Search strategy

The PubMed, Embase, Web of Science, the Cochrane Library, the China National Knowledge Infrastructure (CNKI), Wanfang, and VIP Databases were searched from inception to 1 April, 2021. Search terms included: (“AF” OR “atrial fibrillation”) AND (“ablation” OR “catheter ablation” OR “radiofrequency ablation”) AND (“low-voltage areas” OR “low-voltage zones” OR “low-voltage substrate” OR “LVAs” OR “LVZs” OR “LVS”). We performed a systematic search using population, intervention, comparison, outcomes, study (PICOS) criteria to retrieve all relevant studies. The population of interest included patients with AF who underwent voltage mapping, and the intervention was additional left atrial substrate modification by targeting LVAs. Comparison was performed between study (conventional ablation + LVAs substrate modification) versus control (conventional ablation). The primary outcome was recurrence of arrhythmia, including atrial tachycardia (AT) or AF, and the secondary outcomes contain procedural complications, procedure time, and fluoroscopy time. Studies included randomized controlled trial (RCT) and other trials. Articles following predefined explicit criteria were used: (1) human study and published, (2) all patients performed the left atrial voltage mapping in study, (3) voltage mapping defined LVAs as mapping at sites with voltage <0.5 mV during sinus rhythm, (4) included with and without LVAs ablation in LVAs patients, (5) reported at least one clinical outcome. Exclusion criteria were: (1) conference abstract, (2) degree paper, (3) the study population was not grouped as described, (4) full text was unavailable.

### Data extraction

Two investigators independently screened abstracts and full-text versions of all the studies, and all disagreements were resolved via discussion. We created groups based on characteristics of patients. Patients without LVAs were defined as no-LVAs, LVAs patients with substrate modification were defined as LVAs-ablation, LVAs patients without substrate modification were defined as LVAs-non-ablation. The risk of bias of the randomized control trials was assessed using the Cochrane risk of bias tool, and the seven measures that were graded were as follows: (1) random sequence generation, (2) allocation concealment, (3) blinding of participants and personnel, (4) blinding of outcome assessment, (5) incomplete outcome data, (6) selective reporting, (7) other bias. The risk of bias of a non-randomized study quality was assessed using the Newcastle-Ottawa Scale (NOS) quality assessment scale, and the eight measures that were graded were as follows: (1) representativeness of the cohort, (2) selection of the non-exposed cohort, (3) ascertainment of exposure, (4) outcome absence at start of study, (5) comparability of cohorts, (6) assessment of outcome, (7) adequacy follow-up time, (8) adequacy of follow up of cohorts.

### Statistical analysis

Data analysis was performed using Review Manager (RevMan. Version 5.4. The Cochrane Collaboration, 2020) and Stata/IC 15.0. The Q test was used to test the heterogeneity: *P* ≥ 0.1 and *I*^2^ < 50% suggested homogeneity between studies, and if *P* < 0.1, *I*^2^ > 50% suggested high heterogeneity between studies. To provide more reliable data the random effects model was used in all meta-analyses, and the sensitivity analysis of the index was performed to find the source of heterogeneity. The odds ratio (OR) was calculated and the CI was 95% for dichotomous variables and mean difference (MD), and 95% for continuous variables. *P* values were two tailed, and *P* values <0.05 were considered statistically significant. Continuous variables are not expressed as a mean and standard deviation in literature could be transformed by the formula.^1^

### Trial sequential analysis

Trial sequential analysis (TSA) was performed to analyze the outcomes in order to calculate the required information size (RIS) and correct the risks of type I error. For dichotomous outcomes, the result is conclusive if the cumulative Z-value reaches the TSA threshold or the expected information value. The risk of type I error was maintained at 5% with a power of 80%, and the analysis was performed using the TSA program V.0.9.5.10 Beta.

## Results

### Search result

**FIGURE 1:**
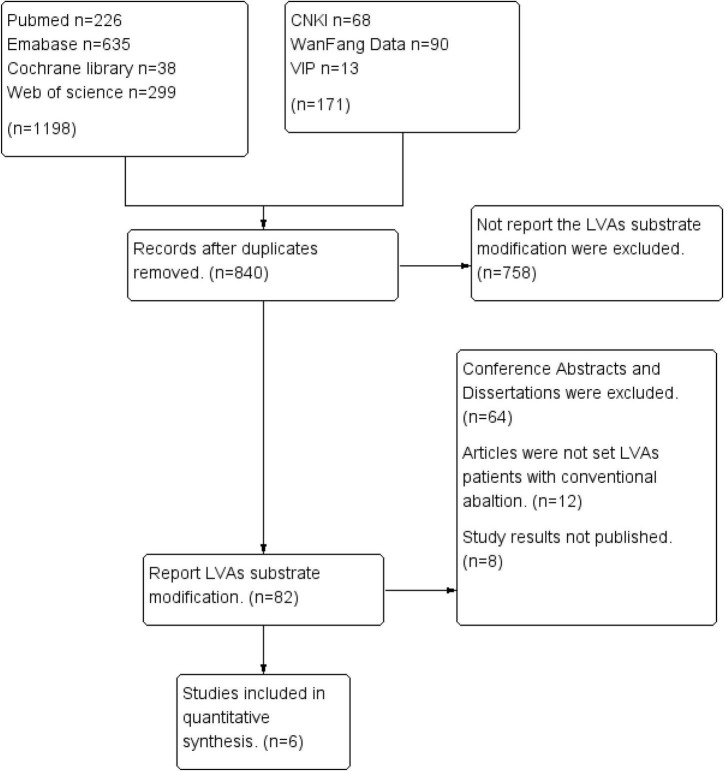
Study selection diagram.

We searched a total of 1,299 reports from all databases, and six studies (16, 17, 19–22) were finally included by excluding duplicates and browsing the abstracts and full text (Figure 1). Of these, two studies were retrospective studies, whose LVAs-non-ablation group was a historical control, three studies were prospective studies and one study was a randomized controlled study.

### Study characteristics and study quality

**FIGURE 2:**
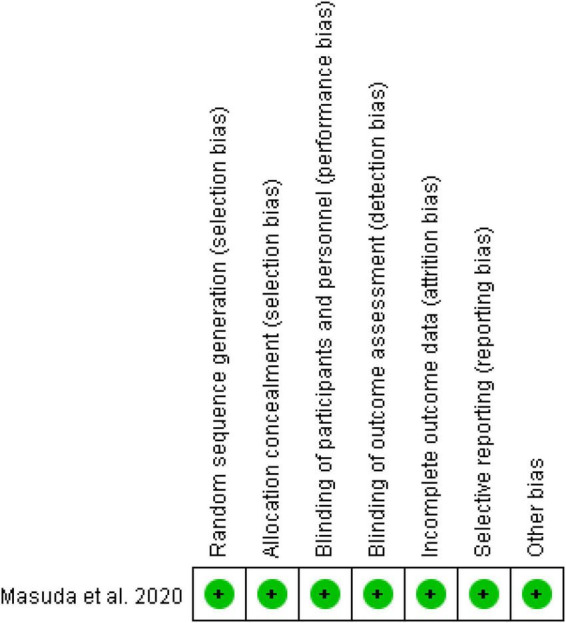
Quality assessment of RCT was evaluated by Cochrane risk of bias tool. The RCT was high-quality.

The characteristics of baseline information for all literature are summarized in Table 1, and the analysis methods and control of potential confounding in the included studies are shown in Supplementary Table 1. A total of 1,175 patients, 712 paroxysmal AF patients and 224 paroxysmal AF patients with LVAs (31.46%), 463 non-paroxysmal AF patients and 230 non-paroxysmal AF patients with LVAs (49.68%), with 257 LVAs patients (with LVAs substrate modification) and 197 LVAs patients (without LVAs substrate modification) were included in the six studies. Other baseline information included gender, age, type of AF, comorbidity, left atrial diameter (LAD), CHA2DS2-VASc score, duration of AF, etc. The characteristics of the procedural information for all literature are summarized in Table 2. Procedural information included the definition of LVAs, procedure strategy, procedural endpoint, blanking period, follow-up survey, follow-up time, etc. The quality of assessment of included studies is shown in Table 3 and Figure 2. One point was deducted because two retrospective studies (19, 20) of the non-exposed population and exposed population were not in the same cohort, and one point was deducted because five cohort studies (17, 19–22) were not adjusted for the most important confounding factors in comparability of cohorts. The bias risk of assessment of RCT was judged to be low.

**TABLE 1:** Characteristics of the included studies.

Study	Study design	Sample size, *n*		Age, y	Male, *n* (%)	Paro xysmal AF, *n* (%)	Persis tent AF, *n* (%)	Long-standing persisitent AF, *n* (%)	Hyper tension	Diabetes melltus	LAD (mm)	CHA2 DS2-VASc	LVEF (%)	AF dura tion, mo
Rolf et al. (19)	Retrospective	no-LVAs	131	59 ± 9	96 (73%)	56 (43%)	75 (57%)	0	91 (75%)	17 (13%)	43 ± 6	NA	60 (55, 62)	66 (24, 110)
		LVAs-ablation	47	67 ± 8	25 (53%)	6 (22%)	41 (78%)	0	40 (85%)	12 (26%)	45 ± 8	NA	60 (50, 63)	35 (16, 90)
		LVAs-non-abaltion	26	67 ± 9	15 (58%)	9 (35%)	17 (65%)	0	23 (89%)	4 (15%)	43 ± 6	NA	57 (45, 65)	60 (33, 84)
Yamaguchi et al. (20)	Retrospective	no-LVAs	62	58 ± 10	48 (77%)	0	47 (76%)	15 (24%)	29 (27%)	4 (6%)	41 ± 5	1 (0∼2)	64 ± 8	NA
		LVAs-ablation	39	66 ± 7	24 (62%)	0	21 (54%)	18 (46%)	27 (69%)	6 (15%)	44 ± 6	2 (1∼3)	62 ± 13	NA
		LVAs-non-abaltion	16	60 ± 9	11 (69%)	0	14 (88%)	2 (13%)	13 (81%)	4 (25%)	43 ± 3	3 (1∼3)	64 ± 8	NA
Zhou et al. (22)	Prospective	no-LVAs	35	58.7 ± 10.6	20 (57%)	0	35 (100%)	0	23 (66%)	6 (17%)	37.3 ± 3.7	NA	63 ± 7	27.0 ± 7.3
		LVAs-ablation	34	60.4 ± 10.6	19 (56%)	0	34 (100%)	0	21 (62%)	6 (18%)	37.6 ± 4.6	NA	62 ± 6	28.2 ± 6.9
		LVAs-non-abaltion	29	60.5 ± 9.0	15 (52%)	0	29 (100%)	0	15 (52%)	7 (24%)	38.1 ± 4.4	NA	62 ± 7	28.9 ± 8.8
Zhou et al. (21)	Prospective	no-LVAs	96	61.0 (52.3∼66.8)	54 (56.3%)	96 (100%)	0	0	63 (65.63%)	17 (17.71%)	36 (34∼37)	NA	65 (57∼68)	27.0 ± 7.3
		LVAs-ablation	74	61.5 (56.8∼69.3)	53 (71.6%)	74 (100%)	0	0	49 (66.22%)	13 (17.57)	36 (34∼37.25)	NA	64 (58∼65)	28.2 ± 6.9
		LVAs-non-abaltion	73	60.0 (52.5∼68.0)	40 (54.8%)	73 (100%)	0	0	40 (54.79%)	18 (24.66)	35 (34∼37.5)	NA	65 (58∼66)	28.9 ± 8.8
Kumagai et al. (17)	Prospective	no-LVAs	61	60 ± 10	57 (93%)	0	20 (33%)	41 (67%)	NA	NA	44 ± 5	NA	59 ± 8	NA
		LVAs-ablation	33	65 ± 8	25 (76%)	0	11 (33%)	22 (67%)	NA	NA	46 ± 5	NA	61 ± 7	NA
		LVAs-non-abaltion	21	65 ± 10	14 (67%)	0	9 (43%)	12 (57%)	NA	NA	46 ± 5	NA	61 ± 8	NA
Masuda et al. (16)	Randomized	no-LVAs	336	67.8 ± 11.6	205 (61%)	336 (100%)	0	0	195 (58%)	51 (15%)	37 ± 6	2.4 ± 1.4	66 ± 9	6 (2, 35)
		LVAs-ablation	30	75.3 ± 7.2	9 (30%)	30 (100%)	0	0	20 (67%)	10 (33%)	40 ± 6	3.6 ± 1.2	64 ± 14	4 (2, 14)
		LVAs-non-abaltion	32	74.7 ± 8.0	9 (28%)	32 (100%)	0	0	16 (50%)	6 (19%)	38 ± 5	3.3 ± 1.3	65 ± 10	5 (2, 23)

LVAs, low voltage areas; AF, atrial fibrillation; LAD, left atrial diameter; LVEF, left ventricular ejection fraction.

**TABLE 2:** Detailed procedures of included trials.

Study	Rolf et al. (19)	Yamaguchi et al. (20)	Zhou et al. (22)	Zhou et al. (21)	Kumagai et al. (17)	Masuda et al. (16)
During the mapping of voltage	SR	SR	SR	SR	SR	SR
Definition of LVAs	>0.5 mV = healthy; 0.2 to 0.5 mV = diseased; <0.2 mV = likely scar tissue; ≥3 adiacent low-volatge point <0.5 mV	<0.5 mV and covering >5% LA surface areas = LVA; <0.1 mV = scar areas.	<0.05 mV = scar areas; <0.5 mV = LVAs	<0.1 mV = scar areas; 0.1∼0.4 mV = LVAs; 0.4∼1.3 mV = transition areas; >1.3 mV = healthy myocardial areas	<0.5 mV and covering >5% LA surface areas = LVAs	<0.5 mV and covering >5 cm^2^ = LVAs
Ablation strategy and LVAs ablation	PVI. Homogenization of LVAs or when not accomplished, linear lesions connect non-conducting tissues and other non-conducting anatomical structures travers target LVAs, or surround large LVAs.	PVI. Non-PV trigger ablation. SVC and CTI physcian’s discretion. All LVAs ablated aiming at homogenization. Futher linear lesion to connect LVAs to anatomical obstacles.	PVI. Anterior LVAs ablated and connected to mitral valve annulus and pulmonary veins. Box iaolation the posterior LVAs.	PVI. Box isolation the LVAs.	PVI. Box isolation the posterior during AF. SVC isolation, CTI isolation, mitral isthums ablation and focal ablation where atrial arrhythmia induced. Box isolation LVAs to connect anatomical obstacles.	PVI. Homogoneously ablated LVAs and posterior LVAs could isolation by PVI, roof and bottom line.
Endpoint of the ablation	PVI: bidirectional conduction block and pace-and-ablate; LVAs: local electrograms, fragmentation, and capture loss; Linear lesion: confirmation of double potentials and analysis of activation sequence.	PVI: bidirectional conduction block; LVAs: homogenization of LVAs and electrogram voltage reduction >50%; LL: creation of double potentials or electrogram voltage reduction of >50%.	PVI: bidirectional conduction block. LVAs: no electrical conduction in all ablated LVAs.	PVI: bidirectional conduction block; box isolation LVAs: ablated to voltage<0.1 mV	PVI: bidirectional conduction block; box isolation the posterior: bidirectional conduction block; LVAs: lack of potential and loss of pacing capture.	PVI: bidirectional conduction block; LVAs: electrogram voltage reduction >50%; LL: bidirectional conduction block.
Monitor of arrhythmia recurrence	Serial 7-day-Holter ECGs at predischagre, 3, 6, and 12 months. Additional Holter or ECGs in case of symptoms.	Visit and ECGs at 2 week, 1 month, and every 3 month. 24-h Holter at 1, 3, and every 6 month.	Visit and ECG or 24 h-Holter at 1, 3, 6, and 12 month.	Visit at 1, 3, 6, 12 month and ECGs once a month within 3 months and Hoter once a month from 3 to 12 months postopeartively.	Monitor of arrhythmia recurrence every month and a questionnaire survey every 3 months. Visit at 3, 6, 12 months and then every 6 months thereafter. 7-day Holter at 6 and 12 months.	Visit and ECGs at 1, 3, 6, 9, and 12 months. 24 h-Holter at 6 and 12 months.
Definition of arrhythmia recurrence	Documented AT/AF > 30 s.	Documented atrial arrhythmia ≥30 s	Documented AT/AF > 30 s.	Documented AT/AF > 30 s.	Documented atrial arrhythmia >30 s.	Doucumented AF/atrial arrhythmia >30 s.
Definition of blanking period	3-month after the procedure.	3-month after the procedure.	3-month after the procedure.	3-month after the procedure.	3-month after the procedure.	3-month after the procedure.
Follow up time	Minimum of 12 months	Minimum of 9 months and end-up 36 months.	12 months	12 months	above 12 months	12 months

SR, sinus rhythm; LVAs, low voltage areas; PVI, pulmonary vein isolation; SVC, superior vena cava; CTI, cavotricuspid isthmus; LL, linear ablation; AF, atrial fibrillation; AT, atrial tachycardia; ECGs, electrocardiograms.

**TABLE 3:** Quality assessment of cohort study.

Study	Represen tativeness of the cohort	Selection of the non-exposed cohort	Ascertain ment of exposure	Outcome absence at start of study	Compara bility of cohorts	Assessment of outcome	Adequacy follow-up time	Adequacy of follow up of cohorts	Score
Rolf et al. (19)	*		*	*	*	*	*	*	7
Yamaguchi et al. (20)	*		*	*	*	*	*	*	7
Zhou et al. (22)	*	*	*	*	*	*	*	*	8
Zhou et al. (21)	*	*	*	*	*	*	*	*	8
Kumagai et al. (17)	*	*	*	*	*	*	*	*	8

Quality assessment of cohort study was evaluated by Newcastle-Ottawa Scale (NOS) quality assessment scale. All 5 cohort studies were high-quality. *Mean 1 point.

### Arrhythmia recurrence

**FIGURE 3:**
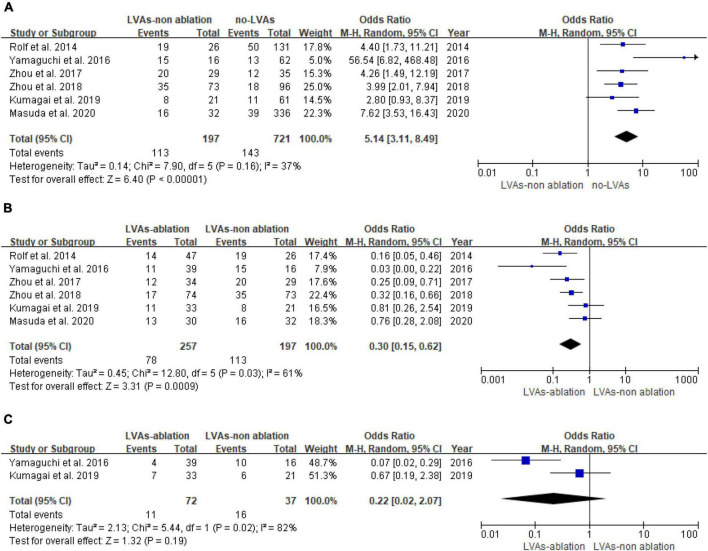
Forest plot of arrhythmia recurrence. **(A)** Recurrence of atrial fibrillation patients with or without LVAs after conventional ablation. **(B)** Recurrence of LVAs patients with or without LVAs substrate modification after first procedure. **(C)** Recurrence of LVAs patients with or without LVAs substrate modification after multiple procedures.

Arrhythmia recurrence is significantly higher in the LVAs group compared with the no-LVAs group in the conventional ablation group (OR: 5.14, 95% CI: [3.11, 8.49]; *P* < 0.00001). Heterogeneity among studies is not significant (*I*^2^ = 37%, *P* = 0.16; Figure 3A). Left atrial substrate modification based on LVAs reduce the arrhythmia recurrence in patients with LVAs (OR: 0.30, 95% CI: [0.15, 0.62]; *P* = 0.0009). There is a moderate degree of heterogeneity (*I*^2^ = 61%, *P* = 0.03, Figure 3B). There is no significant difference in arrhythmia recurrence after multiple procedures (LVAs-ablation group versus LVAs-non-ablation group) (*P* = 0.19, Figure 3C).

### Procedural data

**FIGURE 4:**
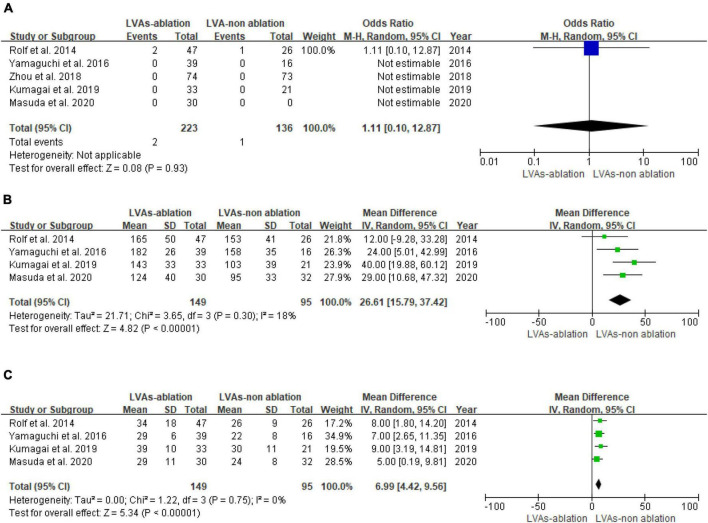
Forest plot of procedural data. **(A)** Complication of LVAs patients with or without LVAs substrate modification. **(B)** Procedure time of LVAs patients with or without LVAs substrate modification. **(C)** Fluoroscopy time of LVAs patients with or without LVAs substrate modification.

The occurrence of ablation complications between the LVAs-non-ablation group and LVAs-ablation group shows no statistical difference (*P* = 0.93, Figure 4A). Substrate modification is associated with higher procedure time (MD: 26.61, 95% CI [15.79, 37.42]; *P* < 0.00001) and higher fluoroscopy time (MD: 6.90, 95% CI [4.34, 9.47]; *P* < 0.00001). Heterogeneity among studies was not significant (*I*^2^ < 50%, *P* ≥ 0.1) (Figures 4B,C).

### Subgroup analysis

We planned several subgroup analyses in advance. The additional LVAs substrate modification reduced the risk of arrhythmia recurrence in LVAs patients whose a follow up time was 12 months (OR: 0.32, 95% CI (0.17, 0.58); *P* = 0.002) and box isolation (OR: 0.37, 95% CI (0.20, 0.69); *P* = 0.002) subgroups, but the type of AF, follow up >12 months, and homogenization subgroups were not statistically significant (Table 4).

**TABLE 4:** Subgroup analysis according to type of AF, follow-up time, type of procedure.

	No. of studies	*I*^2^ (%	OR (95% CI)	*P* value
Total	6	61	0.30 (0.15, 0.62)	0.0009
**Type of AF**				
Paroxysmal AF	2	47	0.46 (0.20, 1.06)	0.07
Non-paroxysmal AF	3	76	0.22 (0.04, 1.08)	0.06
Paroxysmal AF + non-paroxysmal AF	1	NA	0.16 (0.05, 0.46)	0.0007
**Follow-up time**				
12 months	4	39	0.32 (0.17, 0.58)	0.002
>12 months	2	88	0.16 (0.01, 5.28)	0.31
**Type of procedure**				
Homogenization	3	80	0.18 (0.03, 0.96)	0.05
Box isolation	3	21	0.37 (0.20, 0.69)	0.002

### Sensitivity analysis

**FIGURE 5:**
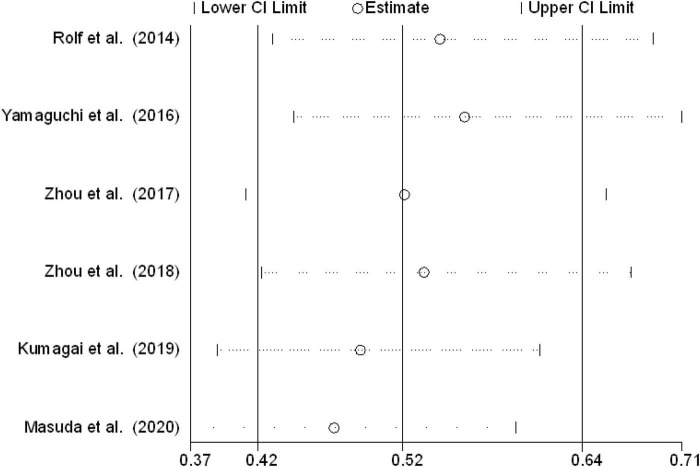
Sensitivity analysis of LVAs ablation in AF patients with LVAs.

Sensitivity analyses were conducted by excluding the included studies one by one. The pooled patients with LVAs underwent substrate modification of results that remained unchanged (Figure 5).

### Trial sequential analysis

**FIGURE 6:**
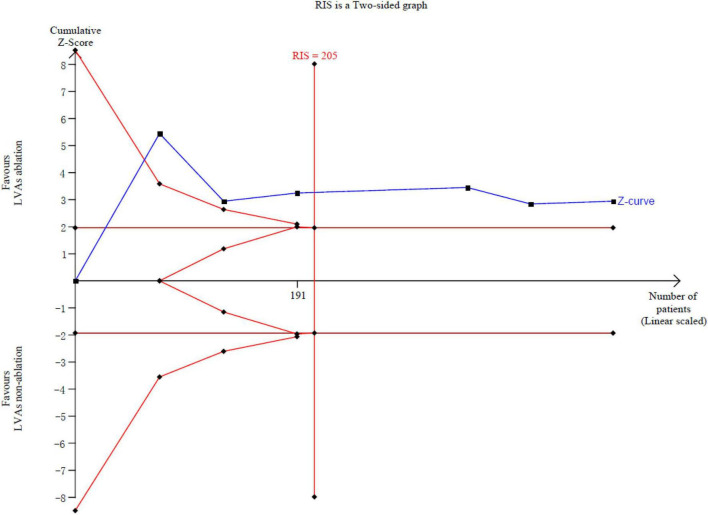
Trial sequential analysis of the LVAs ablation.

TSA software was used for trial sequential analysis. The relative risk reduction was 70%, and the incidence in the control arm was 57%. The results showed that the fourth item of the cumulative Z value crossed the required information size (RIS) value, suggesting that the total clinical efficacy of LVAs ablation in the treatment of AF patients with LVAs has definite evidence and that further research cannot reverse this finding (Figure 6).

## Discussion

This meta-analysis evaluated 1,175 patients from 6 published original articles. To the best of our knowledge, this is the first meta-analysis to evaluate the safety and efficacy of left atrial substrate modification in atrial fibrillation patients with LVAs. In this meta-analysis, the results demonstrated that the recurrence of arrhythmia after ablation was significantly increased in patients with LVAs and additional LVAs ablation after PVI could prove to be effective and safe. However, the effectiveness was limited after multiple procedures. With left atrial substrate modification based on LVAs, the procedure time and fluoroscopy time were increased, but the complication rate was not increased. Compared with the homogenization of the LVAs, box isolation of the LVAs was a better ablation strategy to reduce the arrhythmia recurrence.

### Low-voltage areas as a mark of fibrosis

The mechanisms of AF are complex, including atrial remodeling (structural and electrical remodeling), autonomic nervous system dysfunction, genetic factors, and deregulated calcium homeostasis, etc. (23). Atrial fibrosis is the most predominant characteristic of atrial structural remodeling, linking with all the AF-related mechanisms. Furthermore, many studies have proved that atrial fibrosis is associated with AF recurrence after ablation (24–26). Likewise, many prior studies have demonstrated that patients with LVAs have higher risks of arrhythmia recurrence after ablation than those without LVAs (6, 7), which is similar to our present conclusion. In addition, atrial fibrosis and atrial LVAs have similar upstream factors, such as aging, sex, and atrial size, and the conduction velocity slowing areas are predominantly confined to LVAs, promoting the formation of reentrant (27).

The LGE derived from cardiac magnetic resonance (CMR) remains the gold standard for measuring atrial fibrosis. Oakes et al. examined 81 patients who underwent CMR imaging before the ablation and demonstrated a strong association between LGE and LVAs (28). Spragg et al. found that the LGE of scar imaging agreed with LVAs, and the sensitivity and specificity of LGE for identification of LVAs were 84 and 68% (29), respectively. Nevertheless, another study found some LGE areas were less co-localization with LVAs (30). It may be associated with other tissues that may contribute to reduce the voltage, such as fatty infiltration and amyloidosis.

Despite lack of clear consensus on voltage mapping to identify AF substrates, LVAs, using voltage mapping to describe the areas of scar, are proved to be surrogates for the atrial fibrosis (31–34). In other words, the wider the range of LVAs, the more severe atrial fibrosis and the higher the recurrence rate of arrhythmias. Consistent with the above reports, additional LVAs substrate modification could improve the freedom arrhythmia after conventional ablation.

### Outcomes of low-voltage areas ablation

A recent meta-analysis (35) showed that LVAs modification strategy was superior to traditional ablation strategy, but it was not further explored whether LVAs modification had the same benefit in AF patients with LVAs. Our study further verified that LVAs modification in AF patients with LVAs can further reduce arrhythmia recurrence. As mentioned above, the atrial LVAs, identified on the endocardial voltage map, was correlated with atrial fibrosis and could predict the recurrence after ablation. In this context, many studies have begun to explore the effectiveness of LVAs-guided substrate modification after PVI. Rolf et al. first reported that the AF-free survival was 70 and 67% in the patients with and without LVAs and the success rate in the group of LVAs patients without substrate modification was 27% (19). Subsequently, many researchers investigated the feasibility of LVAs-guided ablation. Most of the research demonstrated a favorable effect on additional LVAs ablation following PVI. However, many operators just performed additional LVAs ablation for all LVAs patients, they did not set PVI alone in LVAs patients as control (12, 36). Finally, our study included six studies which set LVAs patients without substrate modification as the control group, and the outcome of additional LVAs ablation was in agreement with most of the previous studies (OR: 0.30, 95% CI: [0.15, 0.62]; *P* = 0.0009) and related complications were not increased (*P* = 0.93). In contrast, the outcome of multiple procedures did not show a significant difference (study vs. control, *P* = 0.19). This difference of result might be at least partially explained by the small sample size (study vs. control 72 vs. 37), because only two studies are included in this meta-analysis. Platonov et al. had provided histological evidence of a strong correlation between the extent of structural changes with AF duration time and clinical type (25). Unfortunately, no significant difference was found in the AF type subgroup analysis, which may be due to the reduced population after subgroup analysis.

Our results suggested that the outcome of undergoing additional LVAs ablation after PVI is reliable, and we performed a subgroup analysis to further explore the optimal strategy for LVAs ablation. The box isolation of LVAs merits further study. Many strategies have been used for the atrial substrate modification, such as circumferential PVI, linear lesion, complex fractionated atrial electrograms (CFAEs) ablation and LVAs ablation (37). Substrate and Trigger Ablation for Reduction of Atrial Fibrillation II (STAR AF II) trial assigned 589 patients with persistent AF and discovered CFAEs ablation or linear lesion showed less benefit after PVI (38). Kottkamp et al. provided box isolation of fibrotic areas (BIFA), a tailored substrate modification strategy for patients with LVAs, which performs circumferential isolation of the fibrotic areas (37, 39). This strategy was tested and a high success rate was reported by their group in patients with recurrent paroxysmal AF and non-paroxysmal AF (39). STABLE-SR (Electrophysiological Substrate Ablation in the Left Atrium During Sinus Rhythm), a multicenter and randomized clinical trial, enrolled 229 patients with non-paroxysmal and were randomized to study group (conventional ablation + additional substrate modification) or control group (conventional ablation + additional linear lesion). In the study group, patients underwent homogenization and eliminated all tissue of LVAs and CFAEs, respectively. Compared with the control group, there was no significant difference in the success rate after 18 months of follow-up (40). Although two strategies for LVAs substrate modification showed outstanding results, our subgroup analysis demonstrated that the box isolation of the LVAs brings more benefit for LVAs patients. We analyzed the possible reasons for these differences. The homogenization of ablation, endpoint of the reduction in local electrogram region, electrogram amplitude, and loss of capture, did not mean a generation of transmural damage. Furthermore, a half-baked homogenization of the lesion may promote the creation of iatrogenic atrial tachyarrhythmia.

According to the result of the subgroup analysis about follow-up time, the arrhythmia recurrence rate increased when the follow-up interval became longer. In the follow-up time = 12 month subgroup, the additional LVAs substrate modification could reduce the arrhythmia recurrence, however, in the follow-up time >12 month subgroup, additional ablation did not lead to a better result. In our opinions, this phenomenon could be associated with the limited ablation strategy (complete conduction block with transmural lesion creation is difficult to achieve) and age-related atrial fibrosis (41). Furthermore, this outcome could also be related to the characteristics of the population and sample size.

In addition, in terms of safety, although LVAs substrate modification did not increase the resulting complications, the increase in procedure time and fluoroscopy time caused by it should be considered. Recently, the high-power short-duration (HPSD) ablation strategy has gained popularity to improve procedure efficiency (42, 43). This HPSD ablation strategy can be used as a standard approach in LVAs substrate modification in the future.

### Limitation

The strength of this meta-analysis is that we included the control group (conventional ablation) and study group (conventional ablation + additional LVAs ablation) to explore the efficacy and safety of LVAs substrate modification. To figure out the difference of LVAs substrate modification strategy, we performed the subgroup analyses; besides, we conducted sensitivity analyses to strengthen the robustness of the results. However, the results of this study should be interpreted with several potential limitations in mind. First, five of the included studies were retrospective and prospective studies in nature, while there was only one randomized controlled trial and the result of this study has low power for the small sample size. Second, the method for the identification of arrhythmia recurrence varies between studies and this is a major limitation. Third, because of the differences in the type of AF, the degree of ablation, operator expertise, etc., moderate heterogeneity existed among these trial results. Finally, due to the limited number of studies, our findings raise concern about publication bias, which might lead to an overestimation of the pooled effect estimate.

### Conclusions

This meta-analysis has shown that additional LVAs substrate modification after conventional ablation could improve the freedom of arrhythmia recurrence. The box isolation approach appeared more promising. Further large randomized controlled trials are required to confirm these findings.

## Data availability statement

The original contributions presented in the study are included in the article/Supplementary material, further inquiries can be directed to the corresponding author.

## Author contributions

BL designed the study, and reviewed and approved the final manuscript. SM, HF, LW, YW, XW, JZ, BY, YZ, and WZ extracted and analyzed data, and revised the manuscript. SM wrote the manuscript. All authors contributed to the article and approved the submitted version.

## Footnotes

1
https://www.math.hkbu.edu.hk/~tongt/papers/median2mean.html
