# Supplementary material for: A systematic review and meta-analysis of the safety and efficacy of left atrial substrate modification in atrial fibrillation patients with low voltage areas
Source: Front Cardiovasc Med. 2022 Sep 20;9:969475. doi: 10.3389/fcvm.2022.969475 (PMC9530701; doi:10.3389/fcvm.2022.969475)
Supplement: Supplementary file 1 [file Table_1.DOCX]

| Supplementary table 1 | | | | | | | | | |
| --- | --- | --- | --- | --- | --- | --- | --- | --- | --- |
| Study | Free recurrence analysis | Type of regression | Univariate | | | Multivariate | | | Adjusted covariables |
|  |  |  | OR,HR | 95%CI | p value | OR,HR | 95%CI | p value |  |
| Rolf et al. 2014 | K-M curves | Cox | NA | NA | NA | NA | NA | NA | age, sex, AF type, LA appendage flow velocity |
| Yamaguchi et al. 2016 | K-M curves | NA | NA | NA | NA | NA | NA | NA | NA |
| Zhou et al. 2017 | ꭓ² | NA | NA | NA | NA | NA | NA | NA | NA |
| Zhou et al. 2018 | ꭓ² | NA | NA | NA | NA | NA | NA | NA | NA |
| Kumagai et al. 2019 | K-M curves | Cox | NA | NA | NA | NA | NA | NA | NA |
| Masuda et al. 2020 | K-M curves | Cox | HR0.86 | 0.41-1.80 | 0.68 | HR 0.81 | 0.38-1.73 | 0.58 | age, sex, LAD,LVA size |
